# Association between short-term air pollution exposure and the risk of fatal recurrence within 1 year in patients with first-episode acute hemorrhagic stroke: a time-stratified case-crossover study

**DOI:** 10.3389/fpubh.2026.1827069

**Published:** 2026-05-25

**Authors:** Xiaoyong Gu, Jianshu Liu, Hongyu Wang, Dong Hou, Aihua Li, Lu Xu, Jiajia He

**Affiliations:** 1 Zhenjiang Municipal Center for Disease Control and Prevention, Zhenjiang, China; 2 Jiangsu Province Official Hospital, Nanjing, Jiangsu, China

**Keywords:** acute hemorrhagic stroke, first-episode, fatal recurrence within 1 year, short-term exposure, air pollutants

## Abstract

**Objective:**

To analyze the association between short-term exposure to air pollutants and the risk of fatal recurrence within 1 year in patients with first-episode acute hemorrhagic stroke, to provide scientific basis for health management and risk early warning of patients with hemorrhagic stroke in environmental dimension as well as pollution source control.

**Methods:**

Patients with acute hemorrhagic stroke who had their first onset in Zhenjiang City from 2020 to 2023 and experienced fatal recurrence within 1 year were selected as the study subjects. A time-stratified case-crossover study design was adopted, with each case day (the day of stroke fatal recurrence) matched with the date 1 week before, 2 weeks before, and 1 week after the case day as control days. Through conditional logistic regression analysis, the odds ratio of fatal recurrence risk caused by short-term exposure to air pollutants on case days and control days was compared. Based on the model fit results, statistically significant models were identified, followed by analyses of lag effects, dose–response relationships, and interactions among pollutants. Stratification was performed according to sex, age, and season to identify potential effect modifiers of the corresponding variables.

**Results:**

A total of 1,056 case days and 3,168 control days were included in this study. Using China’s national ambient air quality standards as a reference, the average concentrations of PM_2.5_, PM_10_, and O_3__8h in Zhenjiang City were close to the first-level concentration limits, while the concentrations of SO_2_, NO_2_, and CO were far better than the first-level concentration limits. The conditional logistic regression analysis revealed that short-term exposure to PM_2.5_, PM_10_, SO_2_, and NO_2_ was associated with an increased risk of fatal recurrence within 1 year in patients with first-episode acute hemorrhagic stroke (*p* < 0.05), whereas no statistically significant association was observed for CO and O_3__8h (*p* > 0.05). The OR values corresponding to a 1 μg/m3 increase in air pollutant concentration for the 3-day moving average lag (lag02) were as follows: PM_2.5_ (1.010, 95% CI: 1.007–1.013), PM_10_ (1.018, 95% CI: 1.011–1.025), SO_2_ (1.044, 95% CI: 1.012–1.075), and NO_2_ (1.024, 95% CI: 1.013–1.035); for the 2-day lag (lag2), the OR values were PM_10_ (1.013, 95% CI: 1.008–1.017) and NO_2_ (1.017, 95% CI: 1.011–1.023). For each IQR increase in pollutant concentration, the OR value for SO_2_ (1.137, 95% CI: 1.006–1.274) was the highest among the pollutants in the lag02 model, while the OR value for NO_2_ (1.123, 95% CI: 1.021–1.224) was the highest among the pollutants in the lag2 model. The dose–response curves of the 3-day moving average lag (lag02) for all four pollutants were statistically significant at low concentration levels (*p* < 0.05), with the risk of fatal recurrence increasing as the concentration of air pollutants rose. The dose–response curves of PM_10_ and PM_2.5_ with a 2-day lag (lag2) was statistically significant at low concentration levels (*p* < 0.05), with the risk of fatal recurrence first increasing and then decreasing. In contrast, the dose–response curve of NO_2_ with a 2-day lag (lag2) was statistically significant at high concentration levels (*p* < 0.05), showing a rapid upward trend in the risk of fatal recurrence. Female sex, age ≥80 years, and autumn were significant effect modifiers, and PM_10_ attenuated the effect of gaseous pollutants on the risk of fatal recurrence.

**Conclusion:**

Short-term exposure to air PM_2.5_, PM_10_, SO_2_, and NO_2_, even at low concentrations, can increase the risk of fatal recurrence within 1 year in patients with first-episode acute hemorrhagic stroke. Therefore, further strengthening the control and management of pollution sources and establishing tailored risk warning and control measures for different populations and air pollutants are crucial for the health management of current acute hemorrhagic stroke patients.

## Introduction

Stroke, as the second leading cause of death globally, imposes a disease burden of 143 million disability-adjusted life years annually, with hemorrhagic stroke accounting for approximately 37.6% of all stroke cases (1). Studies indicate that stroke patients have a significantly higher risk of recurrence compared to the general population, with a 30-day recurrence risk of 3.1%, a 1-year recurrence risk of 11.1%, and a 5-year recurrence risk of 26.4% (2). Compared to ischemic stroke, patients with hemorrhagic stroke exhibit a higher risk of hemorrhagic recurrence (3, 4) and mortality (5). The recurrence of hemorrhagic stroke has garnered widespread attention due to its extensive cerebrovascular dysfunction, additional physical and cognitive impairments, and extremely high mortality rates (6, 7). Evidences that long-term exposure to air pollutants increases the risk of diseases such as stroke (8–10) and diabetes (11, 12) were convincing. Comprehensive evidence has proved that exposure to air pollution was positively associated with an increased risk of stroke hospital admission, incidence, and mortality (1, 13, 14), and a prior stroke history may enhance susceptibility to air pollutants (15–18). However, existing research lacks evidence linking the fatal recurrent risks of first-time hemorrhagic stroke patients to environmental pollutants, Moreover, the concentrations and weight values of air pollutants involved in different studies vary. Our study focuses on patients with acute hemorrhagic stroke who have their first onset and experience a fatal recurrence within 1 year, selected Zhenjiang city, which has better air pollution control in the Yangtze River Delta region, employing a case-crossover design to analyze the association between short-term exposure to major air pollutants and recurrence mortality risk, aiming to provide scientific basis for health management and risk early warning of patients with hemorrhagic stroke in environmental dimension as well as pollution source control.

## Methods

### Data sources

The incidence data of acute hemorrhagic stroke were sourced from the Zhenjiang Acute Cardiovascular and Cerebrovascular Event Monitoring Database. This monitoring program covered all districts of the city, with the monitoring subjects being local residents aged 18 and above who had resided in the area for at least 6 months. All secondary and above hospitals in the city were required to report cases of acute cerebrovascular events through the Zhenjiang Chronic Disease Network Management Information System within 15 days of discovery. The county/district CDCs conducted quality control reviews of the report card information within 7 days and verified the data monthly against the stroke mortality cases in the China CDC Cause of Death Registry System (19), also supplementary case report work for mortality incidents were conducted. The medical classification of acute hemorrhagic stroke was based on the International Classification of Diseases, 10th Edition (ICD-10), selecting recurrent cases diagnosed with subarachnoid hemorrhage (I60), intracerebral hemorrhage (I61), and other non-traumatic intracranial hemorrhages (I62). Disease diagnosis criteria or definitions refer to appendix 5 in the “China Resident Acute Cardiovascular and Cerebrovascular Event Incidence Monitoring Report” (20). Recurrent cases excluded those that were inactive or in the recovery period, cases of cerebral arteriosclerosis, or cases of non-acute episodes such as outpatient medication or regular hospitalization for “maintenance.” Each episode of the same type was recorded as a case within a 28-day period (21). If another acute episode consistent with diagnostic criteria occurred more than 28 days after onset, it was reported as a recurrent case. If different types of cerebrovascular events occurred within 28 days, they were reported as two separate incidents. Given that the risk of death in stroke patients significantly increases with disease progression (22), this study only included data from hemorrhagic stroke patients who experienced their fatal recurrence within 1 year after the initial onset between 2020 and 2023 for analysis. All fatal episode occurred on the day of recurrence and were verified through the China CDC Cause of Death Registry reporting system, all out-of-hospital deaths were reported as supplementary cases. As this study was an observational study utilizing routine surveillance data, and all patient identifiers had been de-identified prior to data acquisition by the research team, all data used has been anonymized and requires no approval. Therefore, the informed consent process was waived.

During the study, meteorological data were sourced from the Zhenjiang Meteorological Bureau, collecting temperature, relative humidity, and other meteorological data from all monitoring stations in Zhenjiang City, and calculating the arithmetic mean of meteorological data for each jurisdiction. The temperature values were recorded as the atmospheric temperature above 2 meters above ground, with the average temperature calculated at eight time points: 2:00,5:00,8:00,11:00,14:00,17:00,20:00, and 23:00 daily. The concentration data of major air pollutants were obtained from the Air Quality Historical Data Query Platform^1^, collecting 24-h average concentrations of sulfur dioxide (SO_2_), nitrogen dioxide (NO_2_), carbon monoxide (CO), fine particulate matter (PM_2.5_), and inhalable particulate matter (PM_10_), as well as 8-h average ozone concentration (O_3__8h) from all monitoring stations in Zhenjiang City. Meteorological and air pollutant data are complete and continuous, with no missing values. The arithmetic mean of daily pollutant concentrations for each jurisdiction was calculated. The meteorological data and air pollutant concentration level of the local area on the study day and the control day were used as the exposure level of study object.

### Statistical analysis

Continuously variable data with normal distribution were expressed as mean ± standard deviation, while non-normally distributed variables were represented by median and quartiles. Analysis was considered statistically significant when *p* < 0.05. A quality control analysis was conducted using an EXCEL data sheet to organize and analyze the case data of first-episode acute hemorrhagic stroke patients who experienced fatal recurrence within 1 year from 2020 to 2023. The data variables included sex, age, recurrence date, and residential area. A time-stratified case-crossover study design was employed to better control for individual characteristics and potential confounding factors (23). We focused on the acute event-triggering effect of short-term exposure, when conducting temporal stratification, we also considered the fact that excessively long time intervals may lead to significant variations in the distribution of pollutant concentrations and the levels of meteorological factors. For each case day (the day of stroke fatal recurrence), the exposure levels of air pollutants and meteorological factors were matched with the exposure levels of three control days (the day 1 week prior the case day, the day 2 weeks prior the case day, and the day 1 week after the case day, respectively), we also controlled the seasonal, weekday, and temporal trend effects. A total of 3,168 control days were matched for 1,056 study subjects. Spearman correlation analysis was performed to summarize pairwise correlations between air pollutants. Subsequently, a conditional logistic regression model was constructed to estimate the odds ratio (OR) and the 95% confidence interval (95% CI) of risk for fatal recurrence within 1 year for first-episode acute hemorrhagic stroke patients for each specific increase in air pollutant concentration. Temperature, relative humidity, weekday effects, and public holidays were included as covariates in the model, both temperature and relative humidity variables were in the form of natural spline functions [both the degrees of freedom (df) and Knots values were set to 3]. When plotting the dose–response curve, the natural spline function degrees of freedom (df) were set to 3. According to the model fit results, The single—day lag effect of pollutants peaks in the 2—day lag model (lag2), while the cumulative lag effect reaches its maximum in the 3—day moving average lag model (lag02). Both demonstrated statistically significant results. Therefore, this study focused on the analysis of lag2 and lag02 model fit results.

Stratified analysis was conducted by sex (male and female), age (18–44 years, 45–64 years, 65–79 years, and ≥80 years), and season (spring: March–May, summer: June–August, autumn: September–November, winter: December–February) to identify potential effect modifiers of the associated variables. Based on pairwise correlation analysis results and conditional logistic regression analysis results, statistically significant pollutants with low or moderate correlations (R ≤ 0.6) were selected to construct multiple two-pollutant models, sensitivity analysis was conducted by separately adjusting covariate effects such as temperature and relative humidity, nonlinear effects, and public holidays. Conditional logistic regression analysis was performed using the “survival” package in R software (version 4.5.1).

## Results

### Basic information

This study included a total of 1,056 case days and 3,168 control days. The mean age at fatal recurrence for the 1,056 study subjects was 75.56 ± 12.55 years, with 559 males (52.90%) and 497 females (47.10%) (Table 1). The average daily concentrations for each pollutant on case days were as follows: PM_2.5_ is 38.45 ± 24.30 μg/m^3^, PM_10_ is 59.17 ± 34.35 μg/m^3^, SO_2_ is 6.58 ± 2.60 μg/m^3^, CO is 0.65 ± 0.18 mg/m^3^, NO_2_ is 29.83 ± 13.81 μg/m^3^, and O_3__8h is 105.41 ± 48.44 μg/m^3^. The average daily concentrations for each pollutant on control days were as follows: PM_2.5_ is 37.51 ± 24.46 μg/m^3^, PM_10_ is 57.90 ± 35.36 μg/m^3^, SO_2_ is 6.50 ± 2.54 μg/m^3^, CO is 0.65 ± 0.24 mg/m^3^, NO_2_ is 29.63 ± 13.93 μg/m^3^, and O_3__8h is 104.02 ± 48.11 μg/m^3^. Taking China’s national ambient air quality standards (24) as a reference, the first—level concentration limit for 24-h average concentration of PM_2.5_, PM_10_, SO_2_, NO_2_, CO and O_3__8h are as follows: 35 μg/m^3^, 50μg/m^3^, 50μg/m^3^, 80μg/m^3^, 4mg/m^3^, 100μg/m^3^, and the second—level concentration limit for 24-h average concentration are 75 μg/m^3^, 150 μg/m^3^, 150 μg/m^3^, 80 μg/m^3^, 4 mg/m^3^, 160 μg/m^3^, respectively. The average concentrations of PM_2.5_, PM_10_, and O_3__8h in Zhenjiang City are close to the first—level concentration limits, which are the standards for nature reserves, scenic spots, and other areas requiring special protection. Meanwhile, the concentrations of SO_2_, NO_2_, and CO are far better than the first—level concentration limits. The air pollutant concentrations and meteorological data on case days and control days are detailed in Table 2. Pairwise correlations among the six air pollutants were observed, and the details are showed in Supplementary Table S1.

**Table 1:** Characteristics of first-episode acute hemorrhagic stroke patients with fatal recurrence within 1 year in Zhenjiang City from 2021 to 2023.

Variable	*N* = 1,056	Percent (%)
Sex		
Male	559	52.90
Female	497	47.10
Age_group		
18–44 years	21	2.00
45–64 years	156	14.80
65–79 years	436	41.30
≥80 years	443	42.00
Season		
Spring	260	24.60
Summer	254	24.10
Autumn	246	23.30
Winter	296	28.00

**Table 2:** Summary of air pollutant concentrations and meteorological data for case days and control days.

Variable	Mean	SD	Percentile			IQR
			P25	P50	P75	
On case days (*n* = 1,056)						
Air pollutant						
PM_2.5_ (μg/m^3^)	38.45	24.30	22.00	32.00	48.00	26.00
PM_10_ (μg/m^3^)	59.17	34.35	35.00	51.00	76.00	41.00
SO_2_ (μg/m^3^)	6.58	2.60	5.00	6.00	8.00	3.00
CO (mg/m^3^)	0.65	0.18	0.50	0.60	0.80	0.30
NO_2_ (μg/m^3^)	29.83	13.81	19.00	26.00	37.00	18.00
O_3__8h (μg/m^3^)	105.41	48.44	70.00	97.00	136.00	66.00
Meteorological condition						
Temprature (°C)	16.77	9.29	8.78	17.00	25.24	16.46
Relative humidity (%)	71.19	16.10	59.28	70.54	83.97	24.69
On control days (*n* = 3,168)						
Air pollutant						
PM_2.5_ (μg/m^3^)	37.51	24.46	21.00	31.00	47.00	26.00
PM_10_ (μg/m^3^)	57.90	35.36	34.00	50.00	74.00	40.00
SO_2_ (μg/m^3^)	6.50	2.54	4.00	6.00	8.00	4.00
CO (mg/m^3^)	0.65	0.24	0.50	0.60	0.80	0.30
NO_2_ (μg/m^3^)	29.63	13.93	19.00	26.00	36.00	17.00
O_3__8h (μg/m^3^)	104.02	48.11	68.25	94.00	133.00	64.75
Meteorological condition						
Temprature (°C)	16.79	9.34	8.73	17.03	25.35	16.62
Relative humidity (%)	71.06	16.43	58.75	71.38	84.00	25.25

### Lag effects of short-term exposure to air pollutants on mortality risk

**Figure 1:**
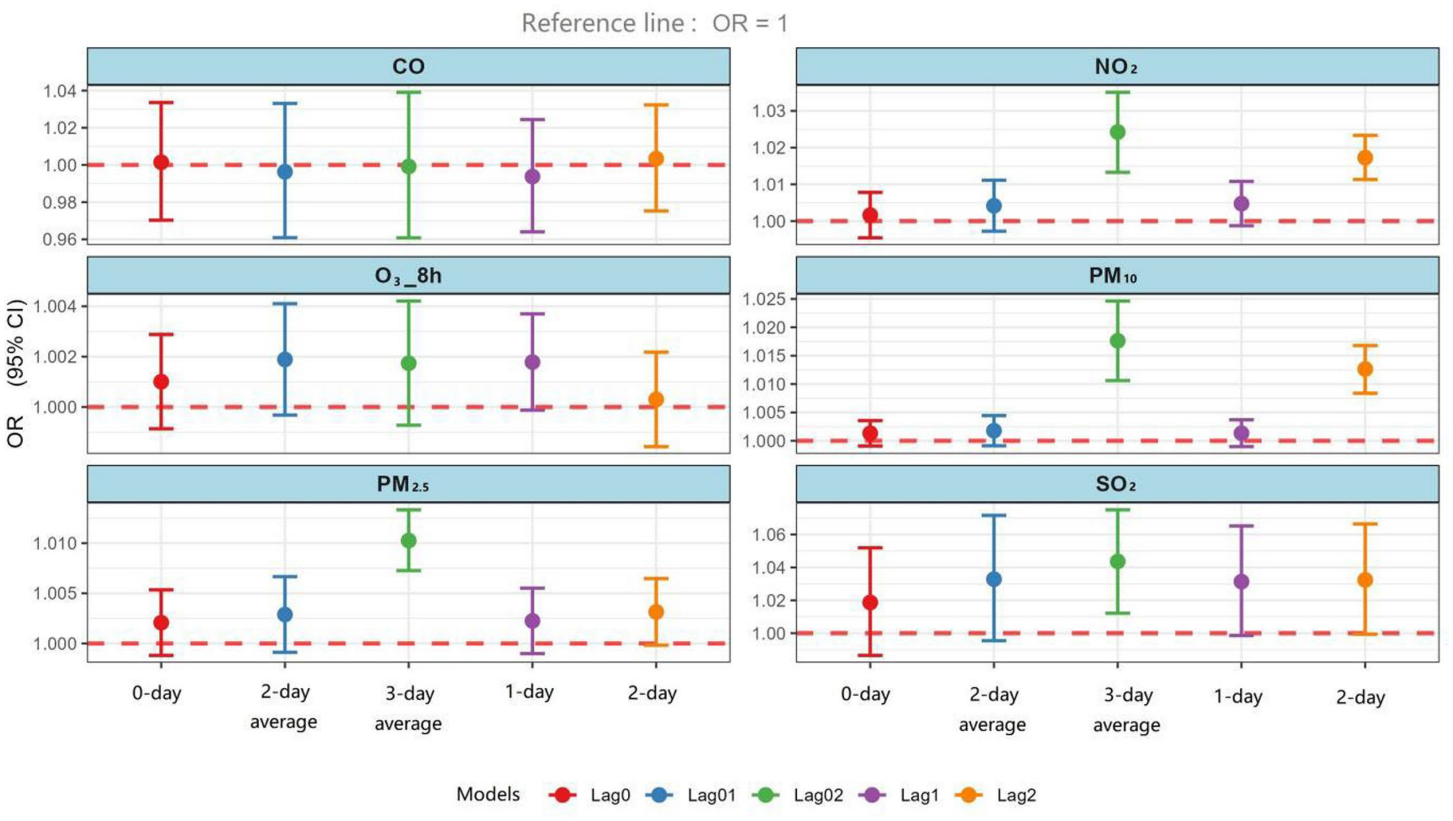
OR values and their 95% CI for the risk of fatal recurrence within 1 year in patients with first-episode acute hemorrhagic stroke for each 1 μg/m^3^ increase in air pollutant concentration.

**Figure 2:**
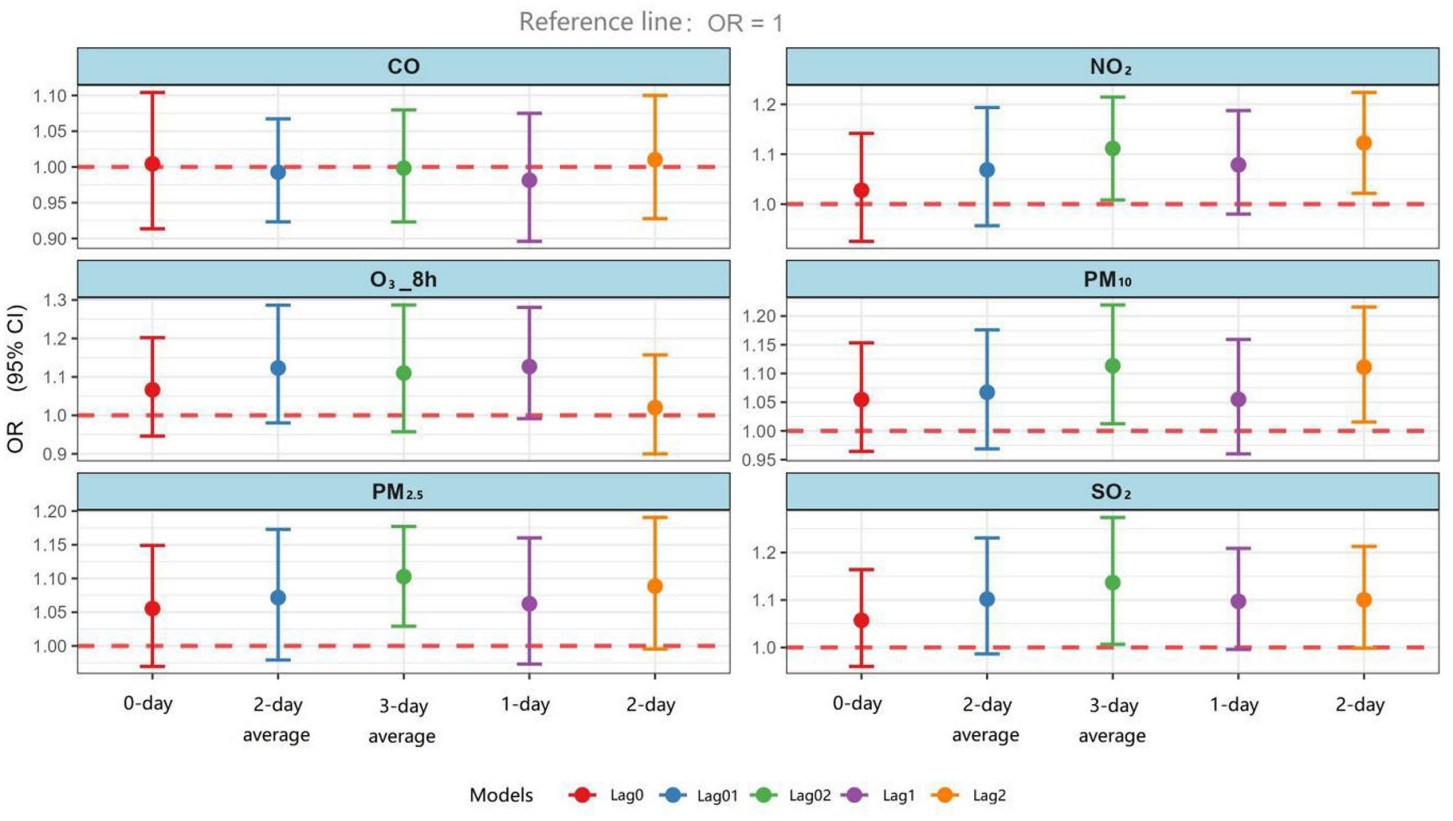
OR values and their 95% CI for the risk of fatal recurrence within 1 year in patients with first-episode acute hemorrhagic stroke for each 1 IQR increase in air pollutant concentration.

Conditional logistic regression analysis revealed that short-term exposure to PM_2.5_, PM_10_, SO_2_, and NO_2_ was associated with an increased risk of fatal recurrence within 1 year in patients with first-episode acute hemorrhagic stroke (*p* < 0.05), whereas such associations were not statistically significant for CO and O_3__8h (*p* > 0.05). The 3-day moving average lag (lag02) model fitting results showed that the OR values for each 1 μg/m3 increase in pollutant concentration were: PM_2.5_ 1.010 (95% CI: 1.007–1.013), PM_10_ 1.018 (95% CI: 1.011–1.025), SO_2_ 1.044 (95% CI: 1.012–1.075), and NO_2_ 1.024 (95% CI: 1.013–1.035). The OR values for each interquartile (IQR) increase in pollutant concentration were as follows: PM_2.5_ (1.103, 95% CI: 1.029–1.177), PM_10_ (1.113, 95% CI: 1.012–1.219), SO_2_ (1.137, 95% CI: 1.006–1.274), and NO_2_ (1.112, 95% CI: 1.008–1.214). SO_2_ exhibited the highest effect among the different pollutants. The 2-day lag model analysis showed that the OR values for each 1 μg/m3 increase in pollutant concentration were: PM_2.5_ is 1.003 (95% CI: 1.000–1.007), PM_10_ is 1.013 (95% CI: 1.008–1.017), SO_2_ is 1.032 (95% CI: 0.999–1.066), and NO_2_ is 1.017 (95% CI: 1.011–1.023). The OR values for each IQR increase in pollutant concentration were: PM_2.5_ (1.089, 95% CI: 0.995–1.191), PM_10_ (1.111, 95% CI: 1.016–1.216), SO_2_ (1.100, 95% CI: 0.998–1.213), and NO_2_ (1.123, 95% CI: 1.021–1.224). NO_2_ exhibited the highest effect among the different pollutants. Details are showed in Figures 1, 2 and Supplementary Table S2.

### Dose–response curve of short-term exposure

**Figure 3:**
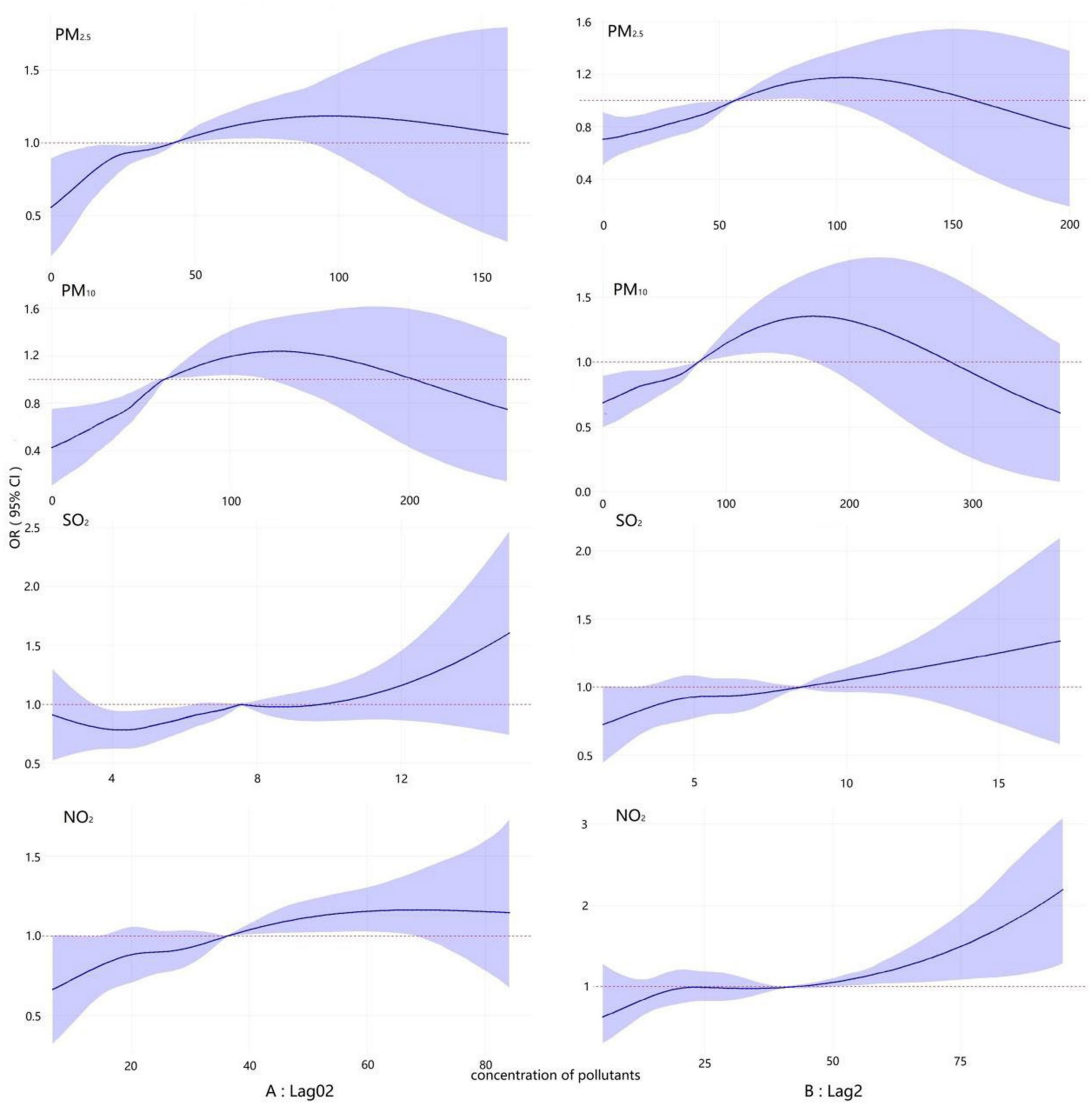
Dose–response curves of short-term exposure to air pollutants (μg/m^3^) and the risk of fatal recurrence within 1 year in patients with first-episode acute hemorrhagic stroke.

The 3-day moving average lag (lag02) exposure dose–response curves for air pollutants revealed that as pollutant concentrations increased, the NO_2_ dose–response curve exhibited an upward trend at low concentrations, followed by a relatively stable level. The SO_2_ dose–response curve initially declined and then gradually increased. The PM_2.5_ and PM_10_ dose–response curves showed rapid increases at low concentrations, followed by a downward trend. All four pollutants’ dose–response curves demonstrated statistically significant at low concentrations in the 3—day moving average (lag02) model (*p* < 0.05). The 2-day lag (lag2) exposure dose–response curves indicated that as pollutant concentrations increased, the effect of PM_10_ and PM_2.5_ first rose and then declined, while the NO_2_ curve initially increased, stabilized, and then rose rapidly. The 2-day lag (lag2) exposure dose–response curves of PM_10_ and PM_2.5_ showed statistically significant effects at low concentrations (*p* < 0.05), whereas NO_2_ curve exhibited statistical significance at high concentrations (p < 0.05), details are showed in Figure 3).

### Stratified analysis results of short-term exposure

Stratified analysis by sex, age, and season revealed that in the 3-day moving average lag (lag02) model, the increased risk of fatal recurrence within 1 year in patients with first-episode acute hemorrhagic stroke was statistically associated with short-term exposure to PM_10_, SO_2_, and NO_2_ in autumn (*p* < 0.05), with short-term exposure to PM_2.5_ and PM_10_ for women (*p* < 0.05), and with short-term exposure to SO_2_ in the 45–69 years and ≥80 years age groups (*p* < 0.05). In the 2-day lag (lag2) model, the increased risk of fatal recurrence within 1 year in patients with first-episode acute hemorrhagic stroke was statistically associated with short-term exposure to NO_2_ in autumn (*p* < 0.05), with short-term exposure to PM_10_ and NO_2_ for women (*p* < 0.05), and with short-term exposure to SO_2_ and NO_2_ in the ≥80 years age group (*p* < 0.05), details are showed in Table 3.

**Table 3:** Stratified analysis results of the association between short-term exposure to air pollutants and the risk of fatal recurrence.

Variable	PM_2.5_		PM_10_		SO_2_		NO_2_	
Lag02	OR (95%CI)	*p-value*	OR (95%CI)	*p-value*	OR (95%CI)	*p-value*	OR (95%CI)	*p-value*
Sex								
Male	1.002 (0.996,1.008)	0.535	1.002 (0.998,1.006)	0.406	1.054 (0.997,1.115)	0.065	1.007 (0.997,1.017)	0.194
Female	1.017 (1.007,1.028)	0.028*	1.023 (1.011,1.034)	0.013*	1.032 (0.973,1.095)	0.293	1.028 (0.997,1.059)	0.175
Age group								
18–44 years	1.004 (0.974,1.034)	0.808	0.999 (0.982,1.017)	0.911	1.009 (0.772,1.320)	0.945	0.998 (0.939,1.062)	0.960
45–64 years	1.008 (0.998,1.018)	0.138	1.004 (0.997,1.011)	0.212	1.112 (1.004,1.232)	0.042*	1.018 (0.998,1.039)	0.080
65–79 years	1.013 (0.996,1.030)	0.359	1.001 (0.997,1.006)	0.526	0.995 (0.933,1.061)	0.872	1.001 (0.989,1.012)	0.908
≥80 years	1.014 (0.997,1.031)	0.269	1.018 (0.999,1.036)	0.079	1.069 (1.004,1.138)	0.037*	1.027 (0.999,1.055)	0.073
Season								
Spring	1.001 (0.991,1.011)	0.825	0.999 (0.994,1.005)	0.781	0.990 (0.918,1.066)	0.785	0.990 (0.970,1.010)	0.333
Summer	0.998 (0.973,1.024)	0.870	0.997 (0.981,1.013)	0.719	1.039 (0.915,1.180)	0.552	1.000 (0.963,1.038)	0.997
Autumn	1.014 (0.999,1.028)	0.072	1.019 (1.011,1.028)	0.025*	1.097 (1.030,1.164)	0.021*	1.034 (1.016,1.051)	0.008*
Winter	1.002 (0.997,1.007)	0.480	1.002 (0.998,1.006)	0.350	1.029 (0.963,1.100)	0.391	1.002 (0.992,1.012)	0.695
Lag2								
Sex								
Male	1.002 (0.998,1.007)	0.359	1.002 (0.999,1.005)	0.132	1.036 (0.991,1.083)	0.115	1.006 (0.998,1.014)	0.160
Female	1.005 (0.999,1.010)	0.077	1.014 (1.005,1.024)	0.023*	1.028 (0.980,1.079)	0.259	1.019 (1.010,1.028)	0.032*
Age group								
18–44 years	1.014 (0.982,1.047)	0.389	1.002 (0.990,1.013)	0.756	0.997 (0.809,1.228)	0.979	1.006 (0.953,1.063)	0.823
45–64 years	1.004 (0.996,1.013)	0.324	1.003 (0.998,1.008)	0.270	1.032 (0.949,1.123)	0.460	1.009 (0.993,1.025)	0.268
65–79 years	1.003 (0.998,1.008)	0.309	1.002 (0.998,1.005)	0.286	1.001 (0.952,1.053)	0.964	1.003 (0.994,1.012)	0.520
≥80 years	1.003 (0.998,1.008)	0.261	1.015 (0.999,1.031)	0.053	1.067 (1.014,1.122)	0.012*	1.031 (1.021,1.041)	0.017*
Season								
Spring	1.001 (0.994,1.009)	0.727	1.001 (0.997,1.004)	0.745	0.980 (0.923,1.041)	0.520	0.997 (0.982,1.013)	0.735
Summer	0.994 (0.975,1.013)	0.527	0.995 (0.983,1.007)	0.431	1.008 (0.915,1.110)	0.876	1.006 (0.979,1.034)	0.682
Autumn	1.004 (0.994,1.014)	0.472	1.005 (0.998,1.012)	0.145	1.057 (0.979,1.141)	0.158	1.022 (1.010,1.034)	0.028*
Winter	1.003 (0.999,1.007)	0.190	1.016 (0.999,1.033)	0.080	1.050 (0.996,1.107)	0.069	1.005 (0.997,1.013)	0.202

*p < 0.05.

### Sensitivity analysis

Based on the results of pollutant correlation analysis, pollutant combinations with low or moderate correlations (*R* ≤ 0.6) were selected to construct two-pollutant models of 3-day moving average lag (lag02) and 2-day lag (lag2), which was used to test model stability. Since O_3__8h and CO were not statistically significant in the model, they were excluded from the sensitivity analysis. After adjusting for temperature, humidity, non-linearity, and week effects in the pollutant model, the OR values for the risk of fatal recurrence within 1 year in patients with first-episode acute hemorrhagic stroke due to short-term exposed to the air pollutants showed no significant changes. The effects of the two-pollutant model were nearly consistent with those of the single-pollutant model. However, it should be noted that adjusting for PM_10_ concentration in the model weakens the effect of gaseous pollutants, which is consistent in both the 3-day moving average lag (lag02) and 2-day lag (lag2) models, details are showed in Supplementary Table S3.

## Discussion

This study employed a time-stratified case-crossover design to analyze the association between short-term exposure to air pollutants and the risk of fatal recurrence within 1 year in patients with first-episode acute hemorrhagic stroke. The mean age of death in the study subjects was 75.56 ± 12.55 years, indicating that the key population at risk for fatal recurrence in acute hemorrhagic stroke patients is the older population. This finding is consistent with our stratified analysis results, which demonstrated that short-term exposure to SO_2_ and NO_2_ significantly increased the risk of fatal recurrence within 1 year in patients with first-episode acute hemorrhagic stroke aged 80 years and above. This may be related to the heightened sensitivity of old hemorrhagic stroke patients to air pollutants (25–27).

The conditional logistic regression analysis revealed that short-term exposure to PM_2.5_, PM_10_, SO_2_, and NO_2_ was associated with an increased risk of fatal recurrence within 1 year in patients with first-episode acute hemorrhagic stroke. Notably, statistical significance was only observed for PM_2.5_ and SO_2_ in the 3-day moving average lag model, while PM_10_ and NO_2_ showed statistical significance in both the 3-day moving average lag model and the 2-day lag model. These findings align with previous studies investigating the association between short-term exposure to air pollutants and the incidence or mortality of hemorrhagic stroke (28–30). Numerous studies (31–34) have reported a positive correlation between the risk of hemorrhagic stroke and concentrations of PM_10_ and NO_2_. Although this differs from the outcome event of fatal recurrence within 1 year in our study, which focuses on first-episode acute hemorrhagic stroke patients, we consider the potential pathogenic mechanisms of PM_10_ and NO_2_ in hemorrhagic stroke—namely, their ability to cause direct ischemic damage to blood vessels, contribute to atherosclerosis and thereby increase the risk of cerebral vascular rupture, and induce vasoconstriction leading to elevated blood pressure (35)—as supporting evidence for our conclusions. Although studies have demonstrated a significant association between short—term exposure to CO and ozone and the risk of hemorrhagic stroke (33–38), no statistically significant association between ozone and CO exposure and fatal recurrence risk was observed in this study. This discrepancy may be attributed to the heterogeneity of the study subjects and the different outcome events being examined. However, we should also note that, Benefiting from effective air pollution control measures, the concentration of air pollutants in our city has remained at a low level, this may have weakened the association between pollutants exposure and the risk of fatal recurrence in patients with acute hemorrhagic stroke. This is also reflected in the fact that the lower limits of the 95% CIs for the OR values of the short-term lag effects of the other five air pollutants, excluding CO, are all close to 1. However, in the lag2 and lag02 models, the statistically significant association we found between short—term exposure to relevant pollutants and an increased risk of fatal recurrence indicates that air pollutants can still exert an impact on hemorrhagic stroke conditions even at low concentrations.

The dose–response curve of short-term pollutant exposure demonstrated that the risk of fatal recurrence within 1 year in patients with first-episode acute hemorrhagic stroke progressively increased with elevated concentrations of PM_2.5_, PM_10_, SO_2_, and NO_2_ in the air. However, three air pollutants showed statistically significant effects only at low concentrations, which was consistent with previous studies (39). In contrast, NO_2_ exhibited statistical significance only at high concentrations, given that this critical concentration (>40 μg/m^3^) corresponds to the 75th percentile of NO_2_ exposure levels in the study subjects, so the actual number of exposure-response relationship observations is relatively sufficient, this conclusion holds practical significance. Previous research (40–42) reported no statistical association between short-term NO_2_ exposure and the risk of stroke, particularly hemorrhagic stroke, which may be related to its health mechanisms of action. Current effective air pollution control has significantly reduced NO_2_ concentrations, making it insufficient to exert health hazards at low levels. As NO_2_ concentrations rise, its role in increasing the risk of fatal recurrence in acute hemorrhagic stroke patients may become more pronounced. This finding underscores the importance of strengthening emission control measures for transportation, a major source of NO_2_ pollution, and highlights the need for further validation in subsequent studies investigating the association between NO_2_ exposure and stroke risk. However, due to the low concentration of pollutants being controlled in this region, it inevitably resulted in fewer observed cases of exposure to high pollutant concentrations. Therefore, the accuracy of the exposure-response relationship under high pollutant concentrations requires further research to confirm Figure 3.

Stratified analysis revealed that the risk of fatal recurrence within 1 year for patients with first-episode acute hemorrhagic stroke who were short-term exposed to air pollutants exhibited sex, age, and seasonal differences, which have been reported in related studies (30, 39, 43, 44). Although the outcome events and exposure lag patterns differed, the disease categories and pollutant types involved remained consistent, indirectly validating the conclusion that we found. This also corroborates the individual-specific differences in this risk (45). Overall, among patients with first-episode acute hemorrhagic stroke, female patients exposed to PM_2.5_, PM_10_, and NO_2_ in the short term, as well as patients aged ≥80 years exposed to SO_2_ and NO_2_ in the short term, exhibited an increased risk of fatal recurrence within 1 year. This may be attributed to differences in the intensity of inflammatory responses (46, 47) induced by air pollution exposure between female and elderly populations, leading to distinct health outcomes.

The stratified analysis also suggests that short-term exposure to PM_10_, SO_2_, and NO_2_ in autumn increases the risk of fatal recurrence within 1 year. A study on seasonal differences between air pollutants and stroke in Changsha, China, which has similar climate conditions to Zhenjiang (48), found that the concentrations of air PM_10_, SO_2_, and NO_2_ in autumn were significantly associated with the risk of hemorrhagic stroke, this study is consistent with ours in terms of region, disease type, and time. This seasonal difference can be explained by the theory regarding the correlation between diurnal temperature variations and human activity intensity (49), the cool autumn climate in this region directly promotes increased frequency and duration of outdoor activities among residents, thereby elevating exposure intensity to air particulate matter and NO_2_, important pollutants in the Yangtze River Delta region (50), combined with SO_2_, under the influence of facilitating factors such as autumn fog and low efficiency of post-growth air phytofiltration (51), leading to health hazards. The analysis revealed that after adjusting for PM_10_ concentration, the effects of SO_2_ and NO_2_, two gaseous pollutants, weakened, this may be attributed to multicollinearity or shared emission sources among pollutants. We should also consider that under different socioeconomic determinants (52), differences in the composition of air pollutants across regions may lead to varying health effects. However, there is no consensus on whether there is an interaction between particulate pollutants and gaseous pollutants in relevant studies (53–55), the interpretation of this effect requires further evaluation based on robust evidence.

Subjects in our study were sourced from the city-wide acute cardiovascular and cerebrovascular event surveillance system, ensuring good representativeness and effectively reflecting the 1-year fatal recurrence of first-episode acute hemorrhagic stroke patients across the city. However, our study has limitations, we used regional average levels of meteorological and air pollutant monitoring data as the expose level of study subjects. The lack of individual exposure data may lead to dilution bias in exposure—response relationships, resulting in either overestimation or underestimation of the corresponding exposure effects. Consequently, the spatial heterogeneity of pollutants and the exposure risks of susceptible populations with specific characteristics cannot be accurately reflected. Future studies should integrate Geographic Information Systems (GIS) and employ high-resolution exposure assessment techniques to obtain precise regional meteorological environmental distribution data. Long-term follow-up tracking of patient health outcomes across different geographical regions should also be conducted. This will provide scientific evidence for stroke patient health management and promote the establishment of an interdepartmental early–warning mechanism for the risk of acute events in stroke patients.

## Data Availability

The original contributions presented in the study are included in the article/Supplementary material, further inquiries can be directed to the corresponding author.

## Footnotes

1
https://www.aqistudy.cn
