# Correction: Association between short-term air pollution exposure and the risk of fatal recurrence within 1 year in patients with first-episode acute hemorrhagic stroke: a time-stratified case-crossover study

**DOI:** 10.3389/fpubh.2026.1898980

**Published:** 2026-07-21

**Authors:** Xiaoyong Gu, Jianshu Liu, Hongyu Wang, Dong Hou, Aihua Li, Lu Xu, Jiajia He

**Affiliations:** 1 Zhenjiang Municipal Center for Disease Control and Prevention, Zhenjiang, Jiangsu, China; 2 Jiangsu Province Official Hospital, Nanjing, Jiangsu, China

**Keywords:** acute hemorrhagic stroke, first-episode, fatal recurrence within 1 year, short-term exposure, air pollutants

Affiliation Jiangsu Province Official Hospital, Nanjing, Jiangsu, China was omitted for author Jianshu Liu. This affiliation has now been added for author Jianshu Liu.

The original version of this article has been updated.

