# Supplementary material for: Association between short-term air pollution exposure and the risk of fatal recurrence within 1 year in patients with first-episode acute hemorrhagic stroke: a time-stratified case-crossover study
Source: Front Public Health. 2026 May 25;14:1827069. doi: 10.3389/fpubh.2026.1827069 (PMC13243267; doi:10.3389/fpubh.2026.1827069)
Supplement: Supplementary file 1 [file Data_Sheet_1.docx]

Supplementary table 1 Pairwise correlation coefficient of air pollutants

| **Pollutants** | PM_2.5_ | PM_10_ | SO_2_ | **CO** | NO_2_ | O_3__8h |
| --- | --- | --- | --- | --- | --- | --- |
| **Lag2** |  |  |  |  |  |  |
| **PM**_2.5_ | 1 | 0.88 | 0.42 | 0.49 | 0.67 | -0.09 |
| **PM**_10_ | 0.88 | 1 | 0.51 | 0.39 | 0.68 | -0.03 |
| **SO**_2_ | 0.42 | 0.51 | 1 | 0.36 | 0.58 | 0.21 |
| **CO** | 0.49 | 0.39 | 0.36 | 1 | 0.49 | 0.13 |
| **NO**_2_ | 0.67 | 0.68 | 0.58 | 0.49 | 1 | -0.14 |
| **O**_3_**_8h** | -0.09 | -0.03 | 0.21 | 0.13 | -0.14 | 1 |
| **Lag02** |  |  |  |  |  |  |
| **PM**_2.5_ | 1 | 0.89 | 0.41 | 0.4 | 0.73 | -0.25 |
| **PM**_10_ | 0.89 | 1 | 0.51 | 0.33 | 0.75 | -0.18 |
| **SO**_2_ | 0.41 | 0.51 | 1 | 0.32 | 0.58 | 0.16 |
| **CO** | 0.4 | 0.33 | 0.32 | 1 | 0.41 | 0.15 |
| **NO**_2_ | 0.73 | 0.75 | 0.58 | 0.41 | 1 | -0.23 |
| **O**_3_**_8h** | -0.25 | -0.18 | 0.16 | 0.15 | -0.23 | 1 |

Supplementary table 2 The lag effect of short-term exposure to air pollutants on the risk of fatal recurrent within 1 year in patients with first-episode acute hemorrhagic stroke

| **Pollutant** | **Lag-day** | **OR(95%CI)** | ***p*-value** |
| --- | --- | --- | --- |
| **PM**_2.5_ | 0 | 1.002(0.999,1.005) | 0.212 |
|  | 1 | 1.002(0.999,1.006) | 0.176 |
|  | 2 | 1.003(1.000,1.006) | 0.063 |
|  | 01 | 1.003(0.999,1.007) | 0.133 |
|  | 02 | 1.010(1.007,1.013) | 0.032 |
| **PM**_10_ | 0 | 1.001(0.999,1.004) | 0.244 |
|  | 1 | 1.001(0.999,1.004) | 0.266 |
|  | 2 | 1.013(1.008,1.017) | 0.022 |
|  | 01 | 1.002(0.999,1.004) | 0.187 |
|  | 02 | 1.018(1.011,1.025) | 0.026 |
| **SO**_2_ | 0 | 1.019(0.987,1.052) | 0.258 |
|  | 1 | 1.031(0.999,1.065) | 0.061 |
|  | 2 | 1.032(0.999,1.066) | 0.054 |
|  | 01 | 1.033(0.995,1.072) | 0.086 |
|  | 02 | 1.044(1.012,1.075) | 0.019 |
| **CO** | 0 | 1.001(0.970,1.034) | 0.929 |
|  | 1 | 0.994(0.964,1.024) | 0.686 |
|  | 2 | 1.003(0.975,1.032) | 0.815 |
|  | 01 | 0.996(0.961,1.033) | 0.842 |
|  | 02 | 0.999(0.961,1.039) | 0.967 |
| **NO**_2_ | 0 | 1.002(0.995,1.008) | 0.610 |
|  | 1 | 1.005(0.999,1.011) | 0.122 |
|  | 2 | 1.017(1.011,1.023) | 0.016 |
|  | 01 | 1.004(0.997,1.011) | 0.240 |
|  | 02 | 1.024(1.013,1.035) | 0.013 |
| **O**_3_**_8h** | 0 | 1.001(0.999,1.003) | 0.292 |
|  | 1 | 1.002(1.000,1.004) | 0.068 |
|  | 2 | 1.000(0.998,1.002) | 0.754 |
|  | 01 | 1.002(1.000,1.004) | 0.094 |
|  | 02 | 1.002(0.999,1.004) | 0.167 |

Supplementary table 3 Results of sensitivity analysis on the short-term exposure lag model for the effects of air pollutants

| **Pollutants** | **Model** | **Lag2** | | **Lag02** | |
| --- | --- | --- | --- | --- | --- |
|  |  | **OR(95%CI)** | ***p*-value** | **OR(95%CI)** | ***p*-value** |
| **PM_2.5_** | Basic | 1.003(0.999,1.007) | 0.063 | 1.01(1.007,1.013) | 0.032 |
|  | Non-linearity | 1.003(0.999,1.007) | 0.086 | 1.01(1.007,1.013) | 0.032 |
|  | Temp-humidity | 1.003(0.999,1.007) | 0.09 | 1.01(1.007,1.013) | 0.023 |
|  | Week effects | 1.003(0.999,1.007) | 0.09 | 1.01(1.007,1.013) | 0.023 |
|  | Control | 1 | NA | 1 | NA |
| **PM_10_** | Basic | 1.013(1.008,1.017) | 0.022 | 1.018(1.011,1.025) | 0.026 |
|  | Non-linearity | 1.013(1.008,1.017) | 0.023 | 1.017(1.01,1.025) | 0.028 |
|  | Temp-humidity | 1.012(1.008,1.017) | 0.026 | 1.017(1.01,1.025) | 0.026 |
|  | Week effects | 1.012(1.008,1.017) | 0.026 | 1.018(1.01,1.025) | 0.026 |
|  | Control | 1 | NA | 1 | NA |
| **SO_2_** | Basic | 1.032(0.999,1.066) | 0.054 | 1.044(1.012,1.075) | 0.019 |
|  | Non-linearity | 1.031(0.997,1.067) | 0.076 | 1.044(1.011,1.078) | 0.016 |
|  | Temp-humidity | 1.028(0.987,1.071) | 0.18 | 1.068(1.011,1.125) | 0.018 |
|  | Week effects | 1.028(0.987,1.071) | 0.18 | 1.068(1.011,1.125) | 0.019 |
|  | Control | 1 | NA | 1 | NA |
| **NO_2_** | Basic | 1.017(1.011,1.023) | 0.016 | 1.024(1.013,1.035) | 0.013 |
|  | Non-linearity | 1.017(1.011,1.024) | 0.023 | 1.024(1.014,1.035) | 0.013 |
|  | Temp-humidity | 1.017(1.011,1.023) | 0.025 | 1.024(1.014,1.035) | 0.013 |
|  | Week effects | 1.017(1.011,1.023) | 0.025 | 1.024(1.014,1.035) | 0.013 |
|  | Control | 1 | NA | 1 | NA |
